# Three-dimensional correlative microscopy of the Drosophila female reproductive tract reveals modes of communication in seminal receptacle sperm storage

**DOI:** 10.1038/s42003-024-05829-y

**Published:** 2024-02-06

**Authors:** Einat Zelinger, Vlad Brumfeld, Katya Rechav, Daniel Waiger, Tally Kossovsky, Yael Heifetz

**Affiliations:** 1 Department of Entomology, The Hebrew University, Rehovot, Israel; 2 Center for Scientific Imaging, The Hebrew University, Rehovot, Israel; 3 Chemical Research Support Department, Weizmann Institute, Rehovot, Israel

**Keywords:** Reproductive biology, Imaging

## Abstract

In many taxa, females store sperm in specialized storage organs. Most insect sperm storage organs have a tubular structure, typically consisting of a central lumen surrounded by epithelial cells. These specialized tubules perform the essential tasks of transporting sperm through the female reproductive tract and supporting long-term sperm survival and function. Little is known about the way in which female sperm storage organs provide an environment conducive to sperm survival. We address this using a combined light microscopy, micro computed tomography (microCT), and Focused Ion Beam Scanning Electron Microscopy (FIB-SEM) approach for high-resolution correlative three-dimensional imaging to advance our understanding of sperm-female interactions in *Drosophila melanogaster*. Using this multimodal approach, we were able to scan the lower female reproductive tract and distal portion of the seminal receptacle at low magnification, and to subsequently zoom in for further analysis on an ultrastructural level. Our findings highlight aspects of the way in which the seminal receptacle keeps sperm viable in the lumen, and set the stage for further studies. The methods developed are suitable not only for Drosophila but also for other organisms with soft, delicate tissues.

## Abstract (web summary)

The anatomy of the Drosophila female lower reproductive tract is probed using a combination of high-resolution correlative three-dimensional imaging techniques, highlighting aspects of seminal receptacle sperm interactions and sperm viability.

## Introduction

**Fig. 1:**
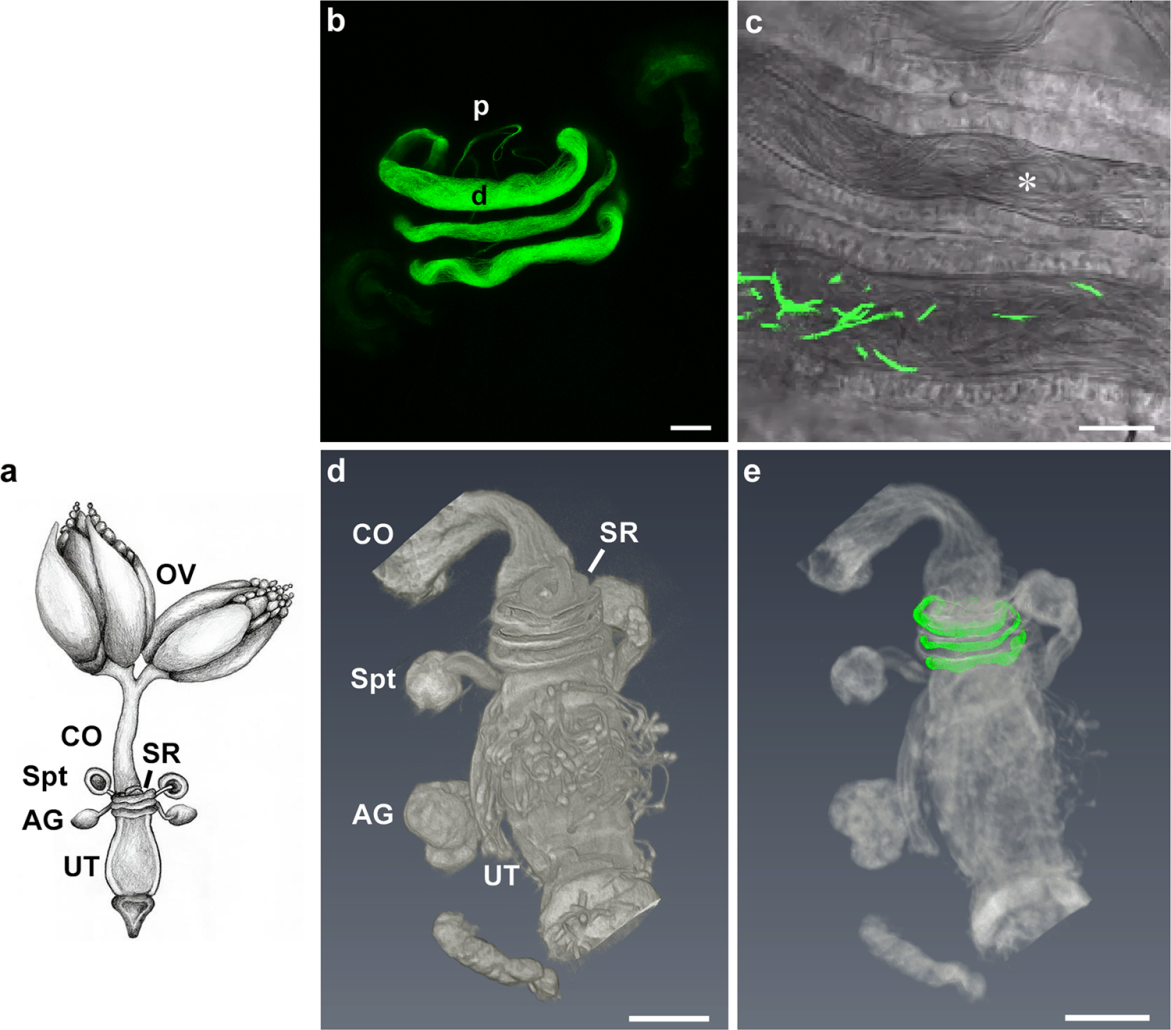
Confocal laser scanning microscopy and micro computed tomography (microCT) visualization of the distal *D. melanogaster* seminal receptacle and the lower reproductive tract. **a** Schematic image of the Drosophila female reproductive tract showing the ovaries (OV), common oviduct (CO), seminal receptacle (SR), a pair of spermathecae (Spt), female accessory glands (AG) and a uterus (UT). **b** Representative confocal maximum projection image of seminal receptacle of 3-day-old female mated to *dj*-GFP male at 24 h post-mating; note the distal (d) and proximal (p) regions of the seminal receptacle. **c** Confocal bright field and fluorescence image of 3-day-old female mated to *pro*-GFP male showing sperm heads (GFP) and tails (bright field, marked by white asterisk) inside the distal seminal receptacle lumen. MicroCT 3D images of the lower reproductive tract (**d**) and a correlative image (microCT and confocal) (**e**) of sperm in the seminal receptacle indicating the position of the distal and proximal regions in relation to the spermatheca and the uterus. Scale bar: **b** = 20 µm; **c**–**e** = 100 µm.

In most animals with internal fertilization, viable sperm is maintained in the body of the female prior to its use in fertilization. In many animals, large numbers of sperm are ejaculated into the female reproductive tract (female RT)^1,2^. Once in the female RT, sperm reach the site of fertilization in the oviduct (e.g. mammals) or the sperm storage organs (e.g. insects) where they can be stored for a few hours to days, months and even several years^2–7^. Mammals store sperm in the vagina, cervix, uterus, or oviduct. Birds and some reptiles typically possess blind-ended specialized sperm storage tubules. Insects have organs called spermathecae and, in some cases, a tubular seminal receptacle, which is connected to the anterior ventral part of the uterus (Fig. 1a) and maintains viable sperm for variable periods before their release for fertilization^8–13^.

During transit in the male and female reproductive tracts, sperm interact with and are modified by the environment. While male factors, such as components of ejaculate, and female factors both influence the initial phase of sperm transfer and storage in the female tract, once the male factors have been processed in the female, the female factors take on a central role in the storage process. Maintenance of viable sperm in the female tract over time is dependent on the female’s capacity to provide a reproductive tract environment conducive to sperm survival^11,14–18^. In many organisms, sperm have a close association with the epithelial cells of the reproductive tract. In mammals, interactions between sperm heads and the surface of the oviduct epithelial cells have been indicated as part of the mechanism prolonging sperm survival in the female reproductive tract^5,19–24^. Direct membrane-to-membrane contact has been shown to be necessary for sperm survival in humans, horses, rabbits and other animals^25–29^. Boar oviduct epithelial cells maintain bonded sperm by suppressing capacitation and motility, thus prolonging sperm lifespan and preserving fertility^30^. Furthermore, the ability to extend sperm viability through incubation with vesicles isolated from the oviduct apical epithelial cells indicates that direct contact and/or vesicle uptake is needed to produce the effect^25–28^. In contrast, the presence of sperm in the oviducts of murine and porcine species alters transcription and translation in oviduct epithelial cells^24,31^; direct communication between sperm and the oviduct epithelium thus affects the activity of both cell types. In other species, sperm are located near the epithelial cells, without directly contacting them. In these cases, the epithelia are involved in maintaining a specific environment around the sperm cells, keeping them viable^10^. Recent studies have shown that secretory oviduct epithelial cells release extracellular vesicles (EVs) into the oviduct lumen^32,33^. EVs are a heterogeneous population of small, cell-derived membrane vesicles released by all cell types; they play crucial roles in both health and disease. EV content (e.g., membrane proteins, nucleic acids, carbohydrates, and lipids), size (e.g. exosomes, microvesicles), quantity, and surface markers are unique, and depend upon the origin of the vesicles^34–38^. EVs have been detected in the oviduct luminal fluid of murine, bovine, human, and other species. These studies showed that oviduct-derived EVs interact with sperm, affecting sperm motility, fertility and viability^30,37–44^.

Organisms such as *Drosophila melanogaster*, which store sperm inside dedicated organs, have evolved cellular specializations including various microvillar and vacuolar domains in the seminal receptacle epithelium, as well as laminar-secreting gland cells in the spermathecae, which release components suitable for sperm maintenance to the microenvironment^12,16,45,46^. Sperm and male accessory gland proteins, transferred to the female during mating, change the gene expression profile in the female and initiate processes that contribute to maintaining sperm in storage^47,48^. Genomic and proteomic studies of female Drosophila have identified molecules which may have roles in sperm storage^49–55^. However, little is known about how the female facilitates sperm storage, or about the coordinated communication networks existing among sperm and organs in the female tract at tissue and molecular levels, which regulate sperm storage.

To gain a deeper understanding of the communication networks enabling sperm to survive for extended periods within the female storage organs, and to elucidate how sperm function is maintained during storage, we first focused on the morphology of the lower reproductive tract and the seminal receptacle. We scanned the lower reproductive tract, as it is an essential part of the sperm’s journey towards the storage organs. Here, we present a combined light microscopy, micro computed tomography (microCT), and Focused Ion Beam Scanning Electron Microscopy (FIB-SEM) approach for high-resolution correlative three-dimensional (3D; volume imaging^56^) to characterize sperm-female interactions in *D. melanogaster*. To this end, we developed a method for high contrast, in-situ, soft tissue visualization, which enabled us to acquire volumetric multiscale data on the anatomy of the lower reproductive tract and the seminal receptacle at high spatial resolution. Furthermore, using a variety of correlative microscopy combinations, we were able to achieve precise correlation within the same region of interest and orient the imaged region of interest at a precise repetitive chosen position. The findings presented here further our understanding of the anatomy of the lower reproductive tract, the distal seminal receptacle epithelium lining the lumen, and the intima layer facing the lumen. Our findings highlight aspects of the way in which the seminal receptacle keeps sperm viable in the lumen, and also uncover possible involvement of EVs in communication between sperm and the distal seminal receptacle. Our multimodal approach made it possible to address questions related to sperm-female interactions at high resolution. Additionally, the methods used in this study are suitable not only for Drosophila, but for other organisms with soft, delicate tissues, as well.

## Results and discussion

### Sperm pass through extensive infoldings and the fertilization chamber in the lower reproductive tract on the way to the storage organs

Females of *D. melanogaster* store ~30–40% of the 4000–5000 sperm transferred during mating^4,57–59^. They can produce 600–800 progeny from the stored sperm over a 3-week period^60–62^. Although more sperm are stored in the seminal receptacle (~400 at maximum) than in the paired spermathecae (~130), and the seminal receptacle is known to be the primary source of sperm used for fertilization^6,16^, the receptacle epithelium has received relatively little attention. The seminal receptacle is a blind-ended tube measuring 2 mm in length, which is coiled against the outer uterine wall^9,63,64^ (Fig. 1). Based on gross morphology, the seminal receptacle is divided into proximal and distal halves; the former includes the entrance to the receptacle and the exit for fertilization, while the latter is the storage site. The two halves of the receptacle differ in lumen size, thickness of the intima, and arrangement of sperm^12,63,65–67^ (Fig. 1b, e). The proximal half of the receptacle has a narrow, slit-like lumen; the distal half is much larger in external diameter and has a wider, ovoid lumen that holds most of the stored sperm (Fig. 1c;^12^). At 24 h post-mating, sperm are organized in the distal seminal receptacle with their heads oriented towards the proximal region (Fig. 1b, c).

To decipher the microscopic anatomy of the lower reproductive tract, and specifically the seminal receptacle, at high resolution, we first spatially mapped the tract in 3D using non-invasive microCT. Since high resolution spatial imaging using microCT requires enhanced contrast of the soft tissues examined, we tested a number of contrasting stains and their combinations. We found that the best contrast was provided by a combination of iodine and tannic acid (Supplementary Note 1, Supplementary Figure 1, Supplementary Table 1). Using this method, we scanned the lower reproductive tract and manually correlated the microCT 3D image with the projection reconstructed confocal fluorescent image of the seminal receptacle. This accurately showed the internal in-situ localization of sperm within the distal region of the seminal receptacle, and enabled further correlative visualization which showed the position of the distal seminal receptacle in relation to the upper uterus (Fig. 1d, e; Supplementary Video 1).

**Fig. 2:**
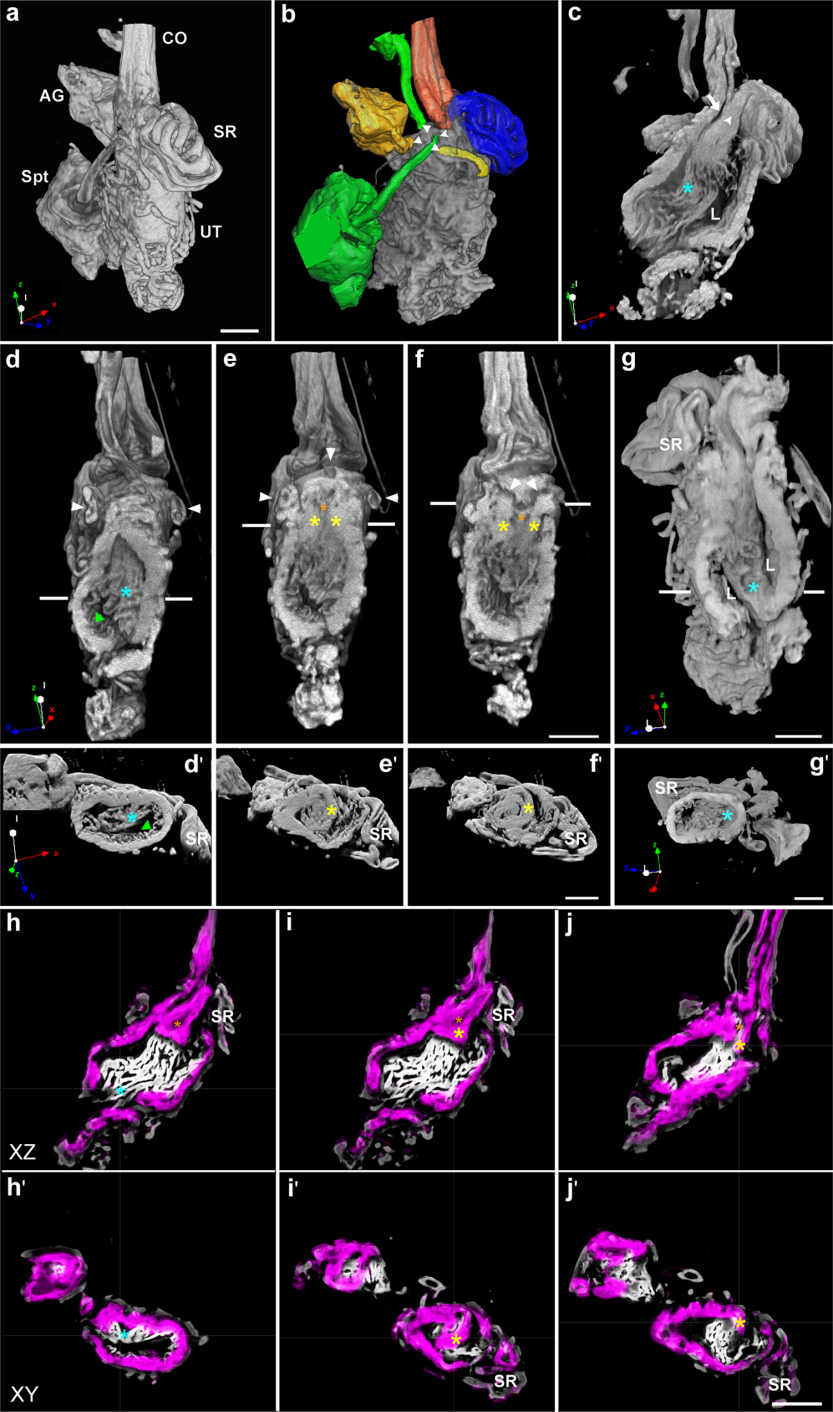
Three-dimensional view of the uterus morphology and the internal connectivity of sperm storage organs and female accessory glands to the uterus. **a**, **b** A microCT volumetric rendering of mated female lower reproductive tract showing the common oviduct (CO), seminal receptacle (SR), spermatheca (Spt) surrounded by fat body (not marked), female accessory gland (AG) and the uterus (UT). Segmentation (**b**) of the sperm storing organs (SR, blue; see also white arrow head shown in **c**; Spt, green) and other organs (AG, yellow; CO, orange) showing their stalks (white arrow heads) opening to a region at the anterior uterus which we term fertilization chamber (orange asterisk shown in **e**, **f**, **h**, **i**, **j**). **c**, **d** show the opening of the common oviduct into the uterus lumen (white arrow in **c**) and internal scans through the uterus, emphasizing the infoldings of the uterine epithelial cells (light blue asterisks, also in **d’**, **g**, **g’ h**, **h’**); lumen, L in **c** and green arrow in **d**. White arrows in **d** indicate the stalks of the spermathecae. White lines in **d–f** indicate the localization of the cross-sections shown in **d’**–**f’**; the boundaries of the fertilization chamber are marked by yellow asterisks that create a narrow space in which the ducts converge (white arrows, **e**, **f**). In Adams and Wolfner^68^, these boundaries are identified as the anterior portion of the papillate elevation at the dorsal side and the anterior uterus projection at the ventral side. **g** Scans through the posterior uterine internal structures showing the lumen (L); white lines indicate the localization of the cross-sections shown in **g’**. Segmentation of the lower reproductive tract reveals the thick multilayer uterine circular muscle fibers (purple) and infoldings of the uterine epithelial cells (white, marked by light blue asterisk) at orthogonal slices (**h**–**j**, at the XZ and the XY axis, **h’**–**j’**). Note that the infolding at the dorsal side is shallower than at the ventral side marked by light blue asterisk **h**, **h’**). Yellow asterisks in **i**, **i’** and **j**, **j’** emphasize the uterine circular muscle fibers in the region of the fertilization chamber marked by orange asterisks in **h**–**j**. The x, y, z vector orientation at the bottom of **a**, **c**, **d**, **g**, **d’**, **g’** shows the position of the uterus. Scale bar: **a**–**f** = 60 µm; **g** = 50 µm; **d’**–**g’**=50 µm; **h**–**j’**=60 µm.

Scans of the entire tract provided additional details of the exterior and interior anatomy of the uterus and connected organs (Fig. 2a). MicroCT volumetric rendering of the mated lower reproductive tract enabled visualization of the microarchitecture of lumens and ducts constituting the seminal receptacle, spermatheca, and female accessory gland, and their connection to the uterus (Supplementary Video 2). The microCT scans also showed the connection of the lower common oviduct to the uterus. To improve the resolution of the connection of the ducts to the uterus, we applied segmentation using Dragonfly software, which partitioned the lower reproductive tract microCT volume into segments corresponding to different tissue/organ classes (Fig. 2b). Interestingly, segmentation of the lower reproductive tract revealed a triangular region in the anterior uterus to which all the ducts are open. We termed this region the “fertilization chamber” (Fig. 2b, c, e, f, h–j marked by arrows, orange asterisk). Adams and Wolfner^68^ describe a pre-oviduct space (POS) in the anterior uterus that forms in mated females between the anterior portion of the papillate elevation, the anterior uterus projection, and the oviduct valve flap. Additionally, Kapelnikov et al. ^52^ highlighted a triangular region in the anterior uterus where the antimicrobial peptide Drosomycin-GFP fluorescence was localized post-mating. Such a triangular region was also detected in the anterior uterus by Schnakenberg and Siegal^13^. We suggest that these regions correspond to the fertilization chamber (Fig. 2h–j), the opening of which enables sperm to reach the sperm storage organs shortly after copulation. Based on Sanchez Lopez et al.^69^, who showed that spermathecal secretory cells secreted GFP to this region, we further hypothesize that the fertilization chamber has an additional function at a later time post-mating. When an egg and sperm arrive in the uterus, secretions derived from the spermathecae and female accessory glands are released into the chamber, plausibly providing a specific pre-fertilization microenvironment for the gametes.

MicroCT scans and segmentation of the microCT volume using ilastik software^70^ further showed extensive folding of the uterine epithelial membrane and muscle layers (Fig. 2d–g, d’–g’, h–j, h’–j’, Supplementary Video 3). Visualization of the muscles encircling the uterus emphasized the oviduct valve flap that separates the common oviduct from the uterus (Fig. 2h–j, h’–j’; marked by yellow asterisk). The analysis also indicates that the depth and spatial density of the membrane folds differ on the dorsal and ventral sides (Fig. 2c–f, d’, h–j, h’, j’ marked by pale blue asterisk; Supplementary Video 2). Extensive infolding and the resulting increase in surface area of the epithelial membrane were detected in the upper oviduct of mated females, while the lower oviduct membrane forms very shallow folds^71^. Infolding of epithelial membranes was also shown to be characteristic of the Drosophila hindgut. The apical and basal plasma membranes of the epithelia in both hindgut regions differ in the depth and spatial density of their membrane folds, and in the abundance of mitochondria associated with membrane infoldings^72^. Folding of the uterine epithelial membrane can tremendously expand its surface area, and this could be particularly important in the mechanism of secretory transport between the epithelial cells and the lumen environment, and for sperm in transit to the storage organs. In addition, expansion of the uterine epithelial folds may assist the passage of sperm and eggs through the lumen, a process requireing flexibility and expansion of the lumen.

### Three-dimensional high z-resolution reconstruction of the distal seminal receptacle provided insights on sperm communication with the luminal epithelium

We used a focused ion beam-milling approach to acquire a higher resolution internal view of the distal seminal receptacle and of the potential contact zone of sperm with the epithelia lining the lumen. FIB-SEM is a dual beam system using a Gallium ion beam to mill nanometric slices from an embedded sample, and an electron beam for SEM imaging. FIB-SEM generates 3D images with high z-resolution isotropic voxels, and enables access to a targeted region with fine resolution, and high precision image quality^73,74^.

**Fig. 3:**
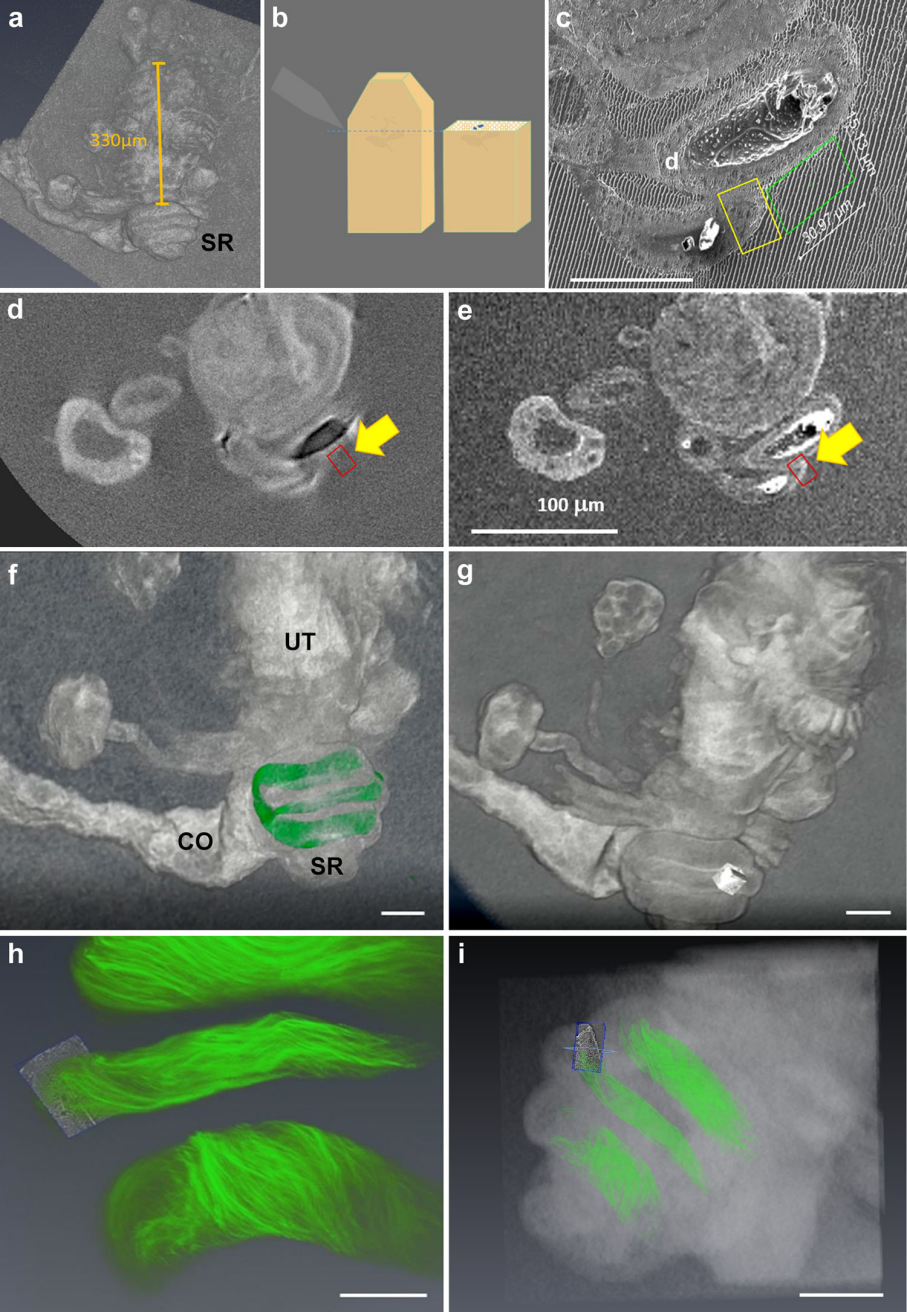
Description of the correlative work flow for milling the precise region of interest using FIB-SEM. **a**, **b** A low magnification microCT image was used to measure the distance needed for trimming the Epon resin block to reach the top of the seminal receptacle as illustrated in **b**. **c** Once trimmed, the top of the resin block was imaged using SEM, and the area to be milled, as well as the trench area was registered; scale bar = 50 µm. To measure the distance from the top of the current surface block to the region of interest for FIB milling, the exact section in the microCT was allocated (**d**) and superimposed with the SEM imaging; scale bar = 100 µm (**e**). To verify that the region of interest in the distal seminal receptacle contained sperm, we also correlated to a confocal image of sperm inside the sampled seminal receptacle with a microCT of the reproductive tract (**f**–**i**); scale bar: **f**, **g** and **i** = 50 µm; **h** = 20 µm. The precise FIB milled region of interest is shown in **g**–**i**; **i** shows the combination of all three 3D modalities (confocal, microCT & FIB-SEM). See also Supplementary Table 2 and Supplementary Video 3.

The FIB-SEM procedure included embedding the lower reproductive tract in Epon resin, followed by microCT scanning of the embedded material to guide milling (Fig. 3a–c). Because iodine is washed out in the embedding process, we used the Osmium-UA common protocol for preparing the sample. Post-embedding, the microCT scan of the Osmium-UA samples displayed lower contrast than the iodine-TA stain, but the scans were suitable for correlation with confocal sperm fluorescence (Fig. 3f). The microCT scans also provided the spatial orientation needed to find the precise region within the distal seminal receptacle of virgin and mated females for FIB milling (Fig. 3h, i, Supplementary Video 3). To that end, a low magnification microCT scan of the Epon embedded samples was acquired for pre-trimming of the block up to the top of the sample. Then a second, higher magnification, microCT scan was performed as close as possible to the destination for start milling, for orientation and accurate trimming (Fig. 3a, b). The precise microCT slice (Fig. 3d) was aligned with the SEM top view image of the trimmed block (Fig. 3c) to identify the position, site and vector for FIB milling (Fig. 3e). For analysis, we milled a 15micron cube of the distal seminal receptacle in 15 nm sections (Fig. 3g–i, Supplementary Video 3).

**Fig. 4:**
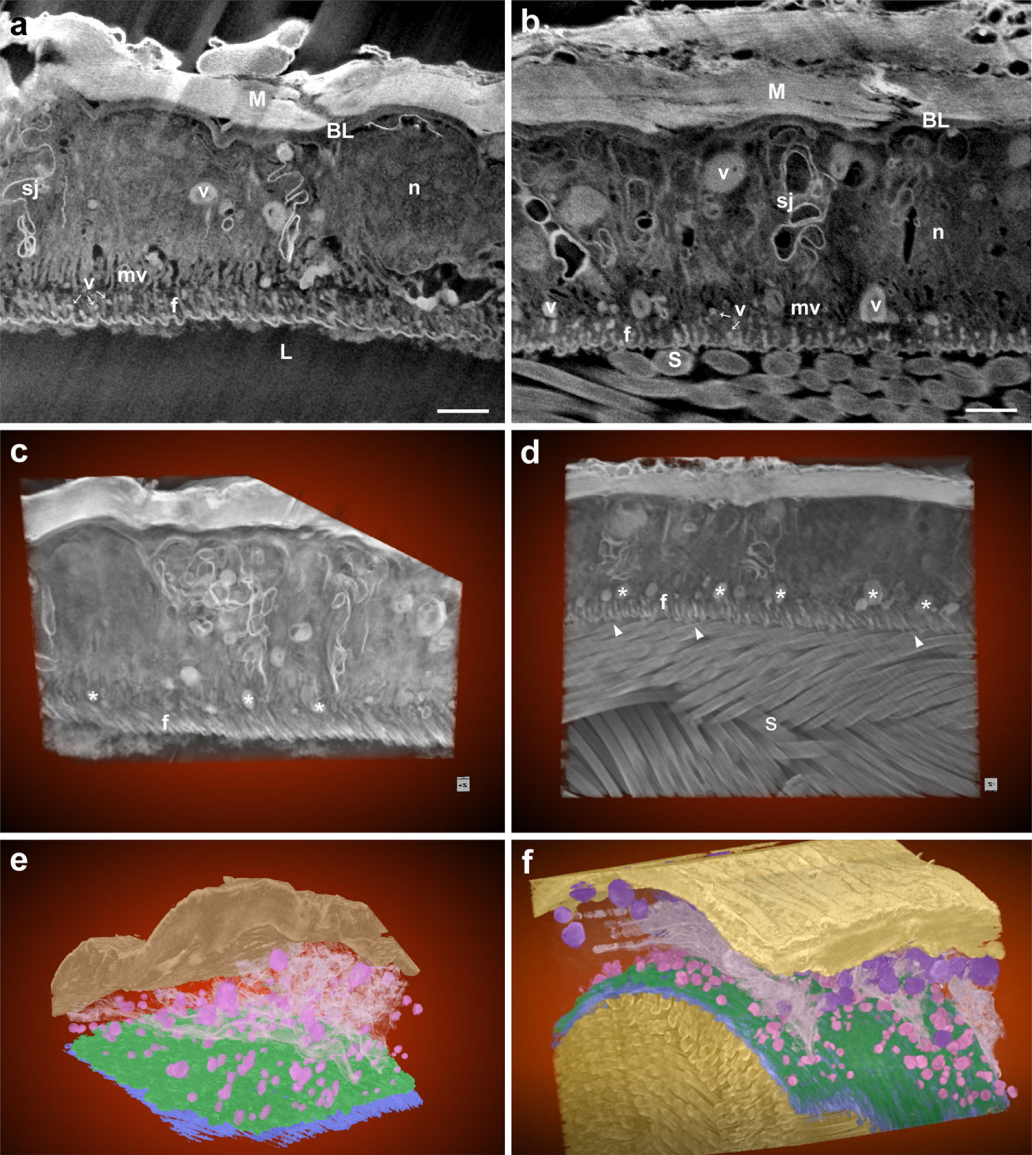
Three-dimensional FIB-SEM projection images of virgin and mated distal seminal receptacle. FIB-SEM 3D projections through the middle tubule of virgin (**a**) and mated (**b**) distal seminal receptacles showing the epithelial cells lining the lumen (L). The apical surface is covered by short microvilli (mv; 100 nm) and lined by an intima. Vesicles (v) are also observed throughout the cytoplasm. Circular muscles (M) surround the basal lamina (BL). Nucleus (n), small vesicles (v, marked by white arrows), fibers comprising the intima layer (f), septate junction (sj), sperm (S); scale bar = 1 µm. 3D projected images clearly show the fiber layer at the epithelium-lumen interface in virgin (**c**) and mated (**d**) distal seminal receptacle. Note the orientation of sperm inside the lumen (**d**, white arrow heads, **f**) and vesicles marked by white asterisk (**c**, **d**). A segmented 3D model of virgin (**e**) and mated (**f**) distal seminal receptacle showing: sperm in the lumen (**f**, dark yellow), corrugated intima facing the lumen (blue) and a fiber layer (green) facing the microvilli, vesicles (purple, pink), septate junction (pale pink), muscle (brownish in **e** and yellow in **f**). The dimensions of the SEM acquired datasets in virgin (**a**) and mated (**b**) samples are shown in Table 1. See also Supplementary Video 4 and 5.

High resolution FIB-SEM revealed that the distal seminal receptacle of virgin and mated females is lined by an epithelium with highly ordered brush border microvilli facing a corrugated intima layer. Septate junctions are present at the cell to cell contacts, and each cell possesses a single large nucleus. The basal lamina is covered by a layer of circular muscle fibers (Fig. 4a–d**;** Supplementary Video 4 and 5). Large numbers of vesicles can be observed throughout the cytoplasm in virgin and mated distal seminal receptacles (Fig. 4). In addition, the orientation of numerous sperm in the lumen appears to be in close interaction with the intima layer (Fig. 4b, d, f; Supplementary Video 5 and 6). Segmentation analysis based on grayscale intensities of the intima layer furthermore indicates that it consists of two fiber layers, one facing the lumen (Fig. 4e, f; blue, corresponding to the corrugated intima) and the other fiber layer facing the microvilli (Fig. 4e, f; green) (Supplementary Video 4 and 5).

**Fig. 5:**
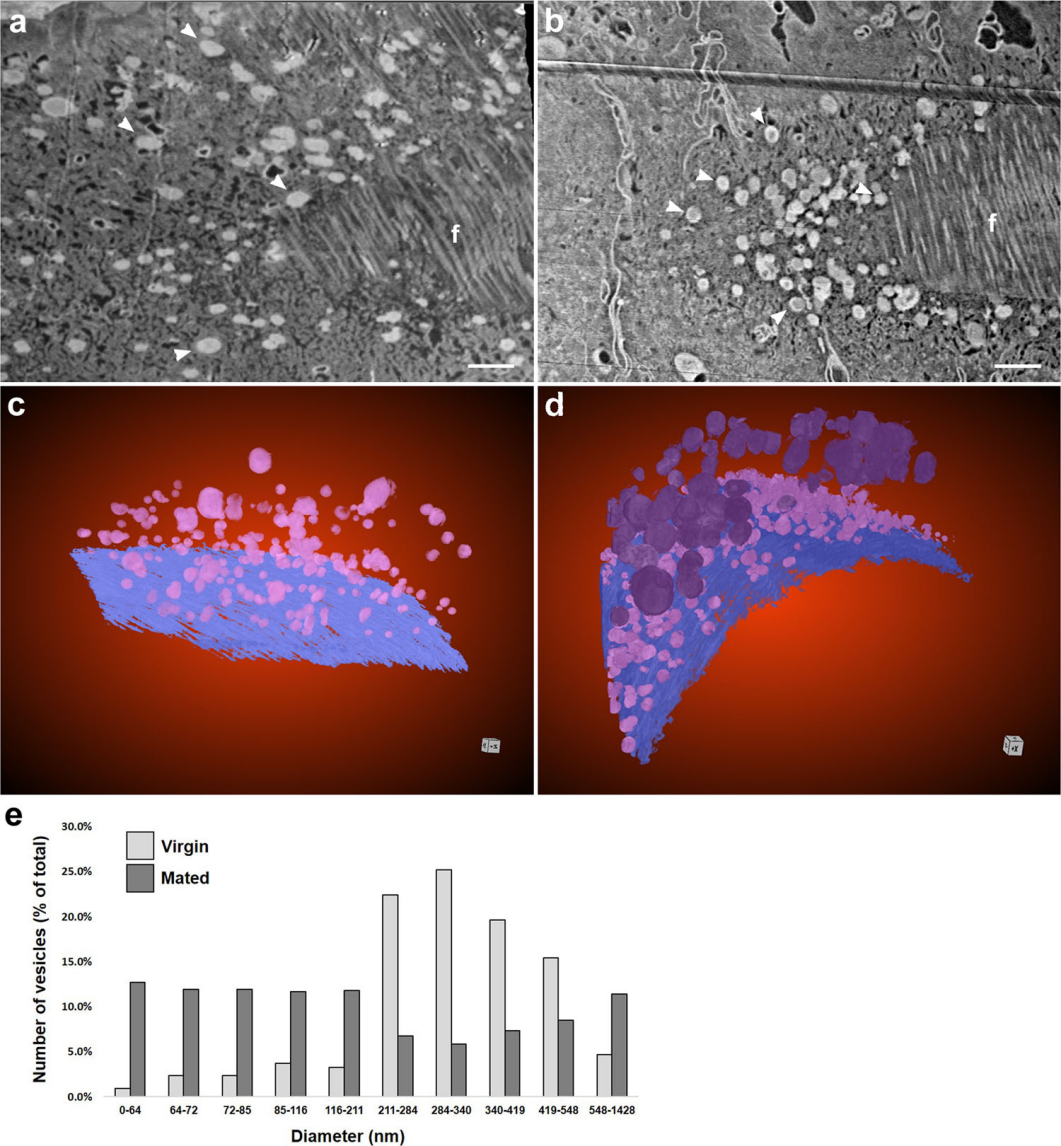
The vesicle population at the distal seminal receptacle changes post-mating. **a**, **b** A cross section of FIB-SEM image at the apical zone showing vesicles dispersed through the indicated zone in virgin (**a**) but densely localize at the apical zone after mating (**b**), white arrow heads; f, fibers at the intima layer; scale bar = 1 µm. Segmented 3D models of vesicles present in virgin (**c**) and mated (**d**) distal seminal receptacles; pink and purple indicate different ranges of vesicle sizes. **e** percentage of vesicles at a particular range of vesicle sizes in virgin (light gray) and mated (dark gray) distal seminal receptacle. Chi-Square test, *p* < 0.0001. See also Supplementary Video 6.

A closer examination of the distal seminal receptacle revealed that after mating, vesicles are trafficked towards the apical and basal zones. These vesicles are surrounded by a lipid bilayer which is characteristics of EVs (Fig. 5a, b). Segmentation of FIB-SEM data followed by vesicle size analysis highlighted a heterogeneous population of vesicles (Fig. 5c, d) ranging from nanovesicles (30–100 nm), small microvesicles (100–200 nm), large microvesicles (200–1000 nm) to large vesicles (>1 µm) (Fig. 5e; Supplementary Video 6). Moreover, while in virgin females the vesicles population comprises mainly large microvesicles (200–550 nm), after mating more nanovesicles and small microvesicles (30–200 nm; Fig. 5e) are present.

**Fig. 6:**
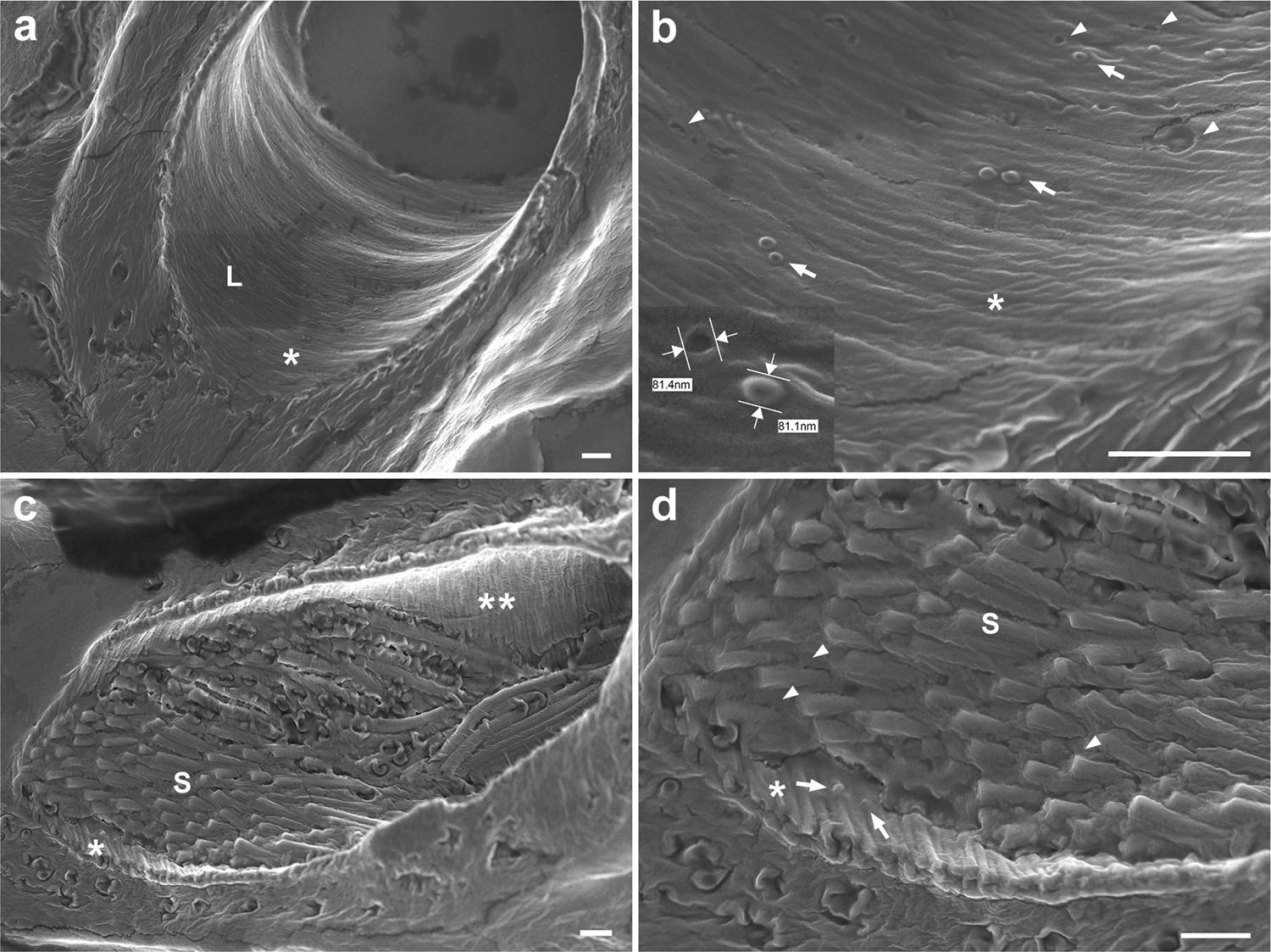
Scanning electron microscopy showing the inner lumen of virgin and mated distal seminal receptacle. 20 µm thick cryostat cross-sections of virgin (**a**, **b**) and mated (**c, d**) distal seminal receptacle. **a** The inner surface of the intima in virgin; L, lumen; corrugated intima marked by white asterisk. **b** Zoom into the intima surface enabled to detect vesicles at a size of 80–100 nm in diameter (see inset, white arrows) on the corrugated intima (marked by white asterisk). In addition, pits at different sizes are visible on the intima (white arrow heads); some of the pits are similar in size to the vesicles. **c** Distal seminal receptacle of mated female filled with sperm (S) showing the corrugated intima marked by white asterisks (internal view, one asterisk; external view, two asterisks). **d** A zoom-in on the intima layer that shows vesicles on the intima close to the sperm (white arrow). The sperm tails (S) that fill the lumen seem to be surrounded by diffuse extracellular material (white arrow head) (**d**). Scale bar = 1 µm.

Visualization of the inner lumen of virgin and mated distal seminal receptacles using 20 µm thick cryostat sections and SEM provided a different perspective of the inner lumen surface (Fig. 6a, c). This enabled the detection of vesicles surrounded by a lipid bilayer inside the lumen, suggesting that EVs are released by the epithelial cells both before and after sperm arrival (Fig. 6b, d, respectively; Supplementary Video 6). Differences in the size of EVs detected pre- and post-mating suggests that the nature of vesicles released to the lumen, and plausibly from the basal zone, changes in the presence of sperm. These seminal receptacle-derived EVs may carry components important for sperm survival in storage. We hypothesize that changes in the EVs population reflect a changing role in the maintenance of sperm in storage. In this context, we note that brush border microvilli and polarized delivery of vesicles to the apical and basal zones are defining features of secretory and absorptive epithelia, like those of the kidney and intestine^75,76^. Though further study is needed, these results suggest that the distal receptacle secretory epithelium changes its secretory mode upon the arrival of sperm to the lumen. A possible role for EVs in maintenance of sperm viability in the reservoir was also suggested by Alcântara-Neto et al. ^77^ for porcine oviductal-derived EVs that interact with sperm.

### The apical surface of the distal seminal receptacle epithelium undergoes changes post-mating

**Fig. 7:**
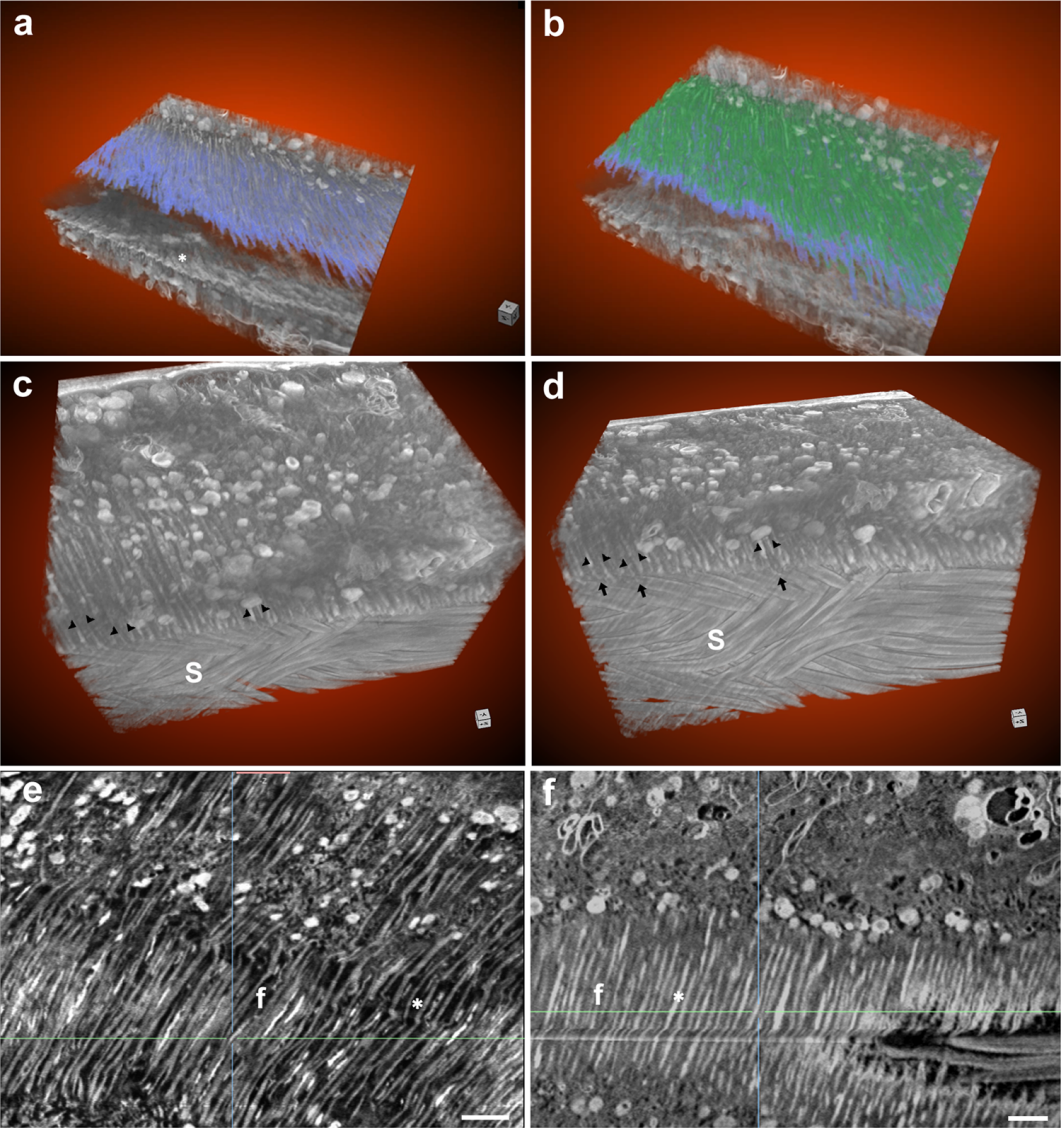
FIB-SEM visualization of the intima fiber layer in virgin and mated distal seminal receptacle. **a**, **b** Segmentation of 3D FIB projection image of virgin distal seminal receptacle is shown. The intima is composed of a corrugated layer marked in blue (**a**, **b**). Unsegmented corrugated intima is also marked by a white asterisk highlighting the intima from the lumen interface perspective (**a**). A second layer of intima depicting parallel fibers is marked in green (**b**). A second layer of intima depicting parallel fibers is also seen in 3D projection of a mated distal seminal receptacle (black arrow heads in a top view **c**, and a top/side tilted view **d**); black arrows in **d** pointing to the grove/wave of the corrugated structure; S, sperm. **e** Two-dimensional FIB image of the intima fibers (f) in virgin, highlight fibers that vary in width. The space between the fibers (marked by white asterisk) appear translucent and uneven in width. **f** In mated, the fibers (f) and the space between the fibers appear more uniform (marked by white asterisk); scale bar = 1 µm.

**Fig. 8:**
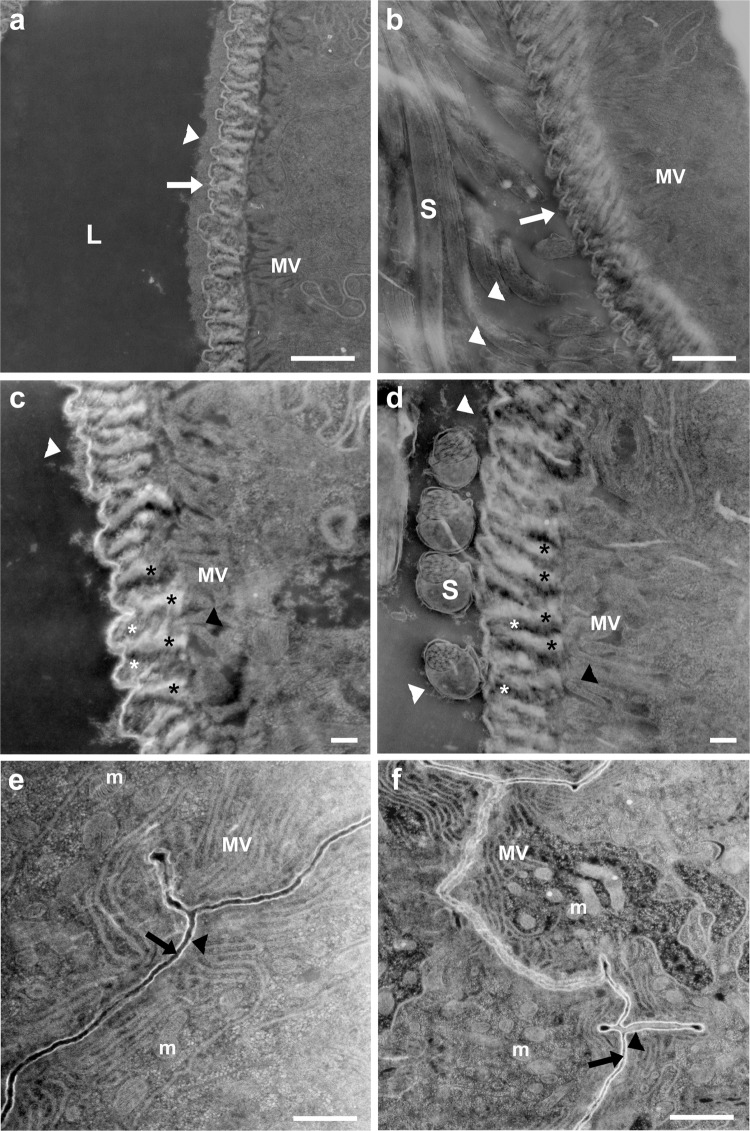
Mating induces changes in the intima layer at the proximal and distal seminal receptacle. Zooming in on the intima at the distal (**a**–**d**) and proximal (**e**, **f**) regions of the seminal receptacle using scanning transmission electron microscopy (STEM in SEM). **a**, **c** View of the corrugated surface of the intima in virgin distal seminal receptacle (white arrow). A layer of electron-dense substance deposited on the intima surface (white arrow). Short brush-border microvilli (MV) are visible at the apical epithelium in both virgin and mated distal seminal receptacle (**a**–**d**); black asterisks mark the ridges/fiber of the corrugated intima in virgin (**c**), and mated (**d**) that are parallel to the microvilli layer (black arrow heads); white asterisks mark the space between the fibers. **b**, **d** In mated, the layer of electron-dense substance deposited on the intima surface is thinner than in virgin (**a**, **c**) white arrow heads. The lumen contains sperm (S) and diffuse extracellular material (white arrow heads) that surrounds the sperm (**b**, **d**). The proximal seminal receptacle epithelia in virgin (**e**) and mated (**f**); microvilli (MV); mitochondria (m); lumen (black arrow); intima (black arrow head). All STEM images are in dark field mode. Scale bar: **a**, **b**, **e**, **f** = 1 µm; **c**, **d** = 200 nm.

**Fig. 9:**
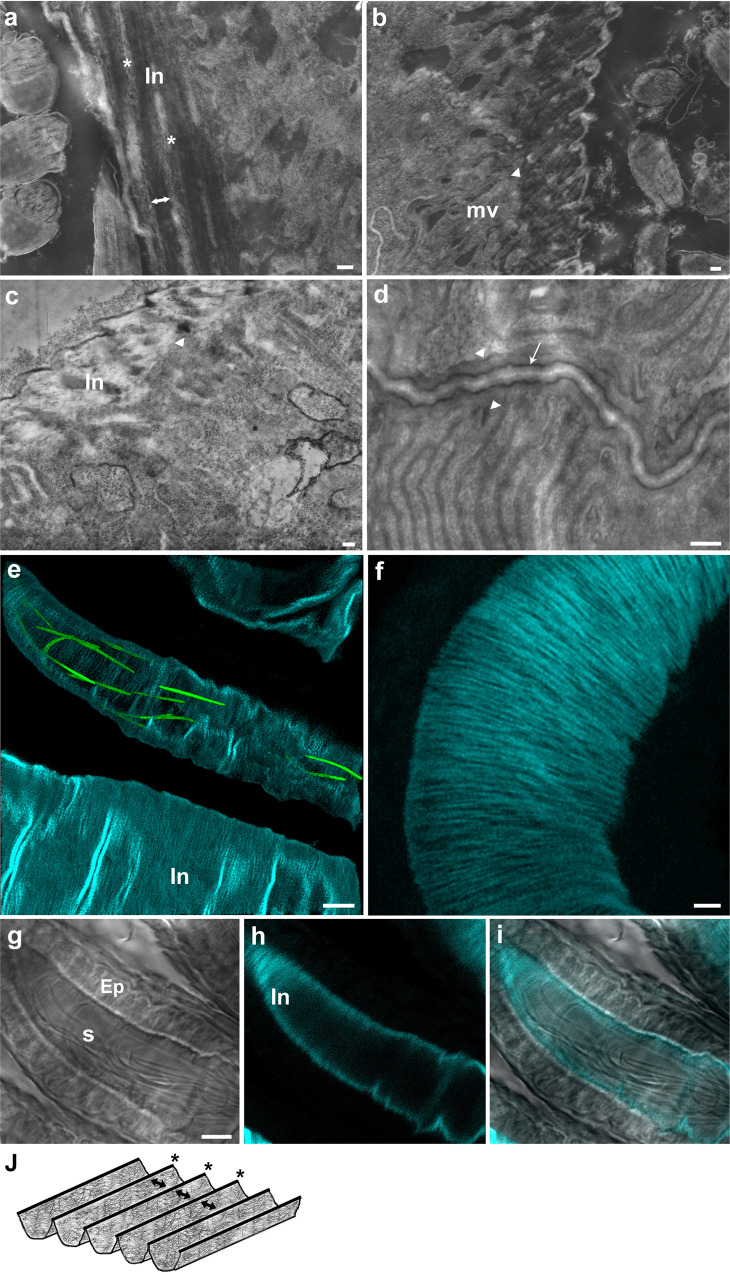
High resolution visualization of seminal receptacle intima layer and chitin fibers. STEM images of distal and proximal seminal receptacle sections (**a**–**d**) and confocal images of distal seminal receptacle (**e**–**i**). Longitudinal (**a**) and transversal (**b**) sections of the distal seminal receptacle in mated showing the nanofiber fiber mesh organization (white asterisks) of the corrugated intima (In); double-ended arrows indicate the grooves between the ridges marked by white asterisks (**a**). Vesicles of various sizes are seen in the lumen of the distal seminal receptacle and in close proximity to the sperm (S) (white arrows) (**b**). The corrugated intima (In) of the virgin seminal receptacle, highlighting the electron-dense substance deposited on the surface and the orientation of the intima in relation to the microvilli (mv) (**c**); the intima is positioned perpendicular to the microvilli (white arrow heads, **b**, **c**). The proximal seminal receptacle thin layer intima (white arrow) facing the thick extracellular matrix (marked by white arrow heads) of the long microvilli (mv) in mated (**d**). **e**–**i** Confocal images of the distal seminal receptacle of female mated to *protamine*-GFP male and stained with Calcofluor white. The organization of the *protamine*-GFP sperm heads in the lumen of the seminal receptacle, that is lined by chitin fibrous intima (cyan) (**e**). The stained intima shows that it consists of chitin fibers (**f**). Bright field image showing sperm tails (s) inside the lumen and the epithelium (Ep) lined by the intima (**g**). A fluorescent image that shows the intima layer stained for chitin and marked by cyan (**h**). An overlay of **g** and **h** emphasizing the chitin based intima (**i**). **j** A schematic drawing showing our proposed organization of chitin fiber mesh of the corrugated intima; double- ended black arrows indicate the grooves between the ridges marked by black asterisks. Scale bar: **a**–**d** = 100 nm; **e**, **g** = 5 µm; **f** = 2 µm.

Three-dimensional reconstruction of the intima layer from FIB-SEM image stacks followed by grayscale segmentation analysis (Fig. 4**;** Fig. 7a–d) and SEM imaging of the intima surface (Fig. 6) suggests that the intima undergoes structural modification prior to the arrival of sperm. To gain further insight into the structure of the intima layer at the apical epithelium and verify the difference in fiber organization, we next focused on the fiber layer facing the microvilli, and analyzed the width of the fibers and the space between them in virgin and mated FIB-SEM samples (Fig. 7e, f). Measurements were performed on aligned 2D images of virgin and mated intima fiber layers (Supplementary Fig. 2a, b). The width of the fibers and space between them were found to be smaller in mated than in virgin samples. Additionally, the width of the fibers and space between them were variable in virgin samples, and more uniform in mated ones (70–120 nm vs. 60–100 nm, and 140–290 nm vs. 60–160 nm, respectively; Supplementary Fig. 2c). High resolution visualization of the intima fiber network using scanning transmission electron microscopy (2D STEM) revealed that the intima does not lie parallel to the microvilli, but is rather positioned perpendicularly (Figs. 8 and 9). The fiber ridges between the grooves are highly aligned in mated as compared to virgin samples (Fig. 8c, d**;** black asterisks), and the intima itself appears to be composed of a very fine mesh of fibers (Fig. 9a, white asterisks, Fig. 9j, black asterisks). Interestingly, the corrugated intima facing the lumen is covered by a homogeneous thick electron-dense extracellular matrix in the virgin, and a thin extracellular matrix in the mated (Fig. 8a, c and 8b, d, respectively). In the proximal seminal receptacle, the lumen is narrow and the intima layer is thin in both virgin and mated (Fig. 8e, f, respectively). Moreover, while the proximal epithelium gives rise to densely packed, long microvilli covered by a thick extracellular matrix, the distal, highly ordered brush border microvilli^12^ are covered by a thin extracellular matrix that is difficult to distinguish in both females (white arrow heads in distal: Fig. 9b, c, respectively; proximal: Fig. 9d). Finally, by using Calcofluor stain to visualize chitin, we show that the intima is composed of chitin, and the stain pattern of the dense uni-axially aligned fibers matches the intima ultrastructure presented here (Fig. 9e–i).

Taken together, at the morphological level, our results provide several lines of evidence suggesting that mating induces ultrastructural changes in the intima of the distal seminal receptacle, and that these changes are consistent with the role of this tubular organ in maintaining sperm viability. These findings are similar to those of Kapelnikov et al. ^71^ who showed that the final maturation of the Drosophila oviduct is triggered in the last differentiation stage by mating. They further proposed that oviduct maturation is characterized by mating-independent and -dependent processes, and that both of these pathways are essential to produce a functional oviduct.

Changes detected in the fiber width and space between the fibers in the intima fiber layer point to alterations in the properties of the layer. Such alterations may, for example, provide protection to the delicate brush border microvilli of the distal seminal receptacle. The intima fiber layer may also constitute a selective permeability barrier to pathogens, and facilitate uptake and/or secretion to and from the receptacle lumen, as was shown for the peritrophic membrane in the midgut^78–80^. The geometric properties of the corrugated intima facing the lumen hint toward a possible biomechanical function essential for keeping sperm viable and functional once in storage. As the seminal receptacle is a long tube closed at its end, once sperm, fluid and other components that arrive with sperm fill the lumen, there is a need to control the pressure and flow to protect sperm. The geometric properties of the corrugated intima, in conjunction with the circular muscle layer at the basal region of the tubule, affect the internal lumen pressure and turbulence, flow rate and other characteristics affecting the sperm. Thus, we hypothesize that the observed post-mating alterations in the geometric properties of the intima are essential for regulating the pressure and turbulence created in the lumen upon sperm arrival. These changes are thus critical for sperm survival in the distal seminal receptacle tubule.

## Conclusion

Despite extensive knowledge and interest in sperm storage in Drosophila and other organisms, little is known about the Drosophila seminal receptacle. The findings presented here advance our understanding of the manner in which the seminal receptacle keeps sperm viable in the lumen.

We have developed a method for visualizing soft tissue via different microscopy modalities. Using five different 2D and 3D modalities (i.e., confocal, microCT, FIB-SEM, SEM, STEM), correlative methods, and various image analyses, we were able to view the lower reproductive tract and internal distal seminal receptacle at high spatial resolution. Specifically, we have begun to understand the structure of the seminal receptacle epithelium lining the lumen and the intima layer facing the lumen. Additionally, we contribute new information on uterus morphology, and possible secretion routes encountered by sperm on their way to the storage site and the site of subsequent fertilization. Focusing on the distal seminal receptacle of virgin and mated females at 24 h post-mating, we detected mating-derived changes in the morphology of the intima layer and secretions. The results highlight the corrugated structure of the intima, and show that mating induces changes in the intima geometric properties. Interestingly, corrugated intima is a conserved structure also seen in other insects, such as, for example, the ductus receptaculi of *Teleogryllus commodus* (Orthoptera)^81^, the seminal receptacle of *Armadillidium granulatum* (Isopoda)^82^, and the spermatheca and seminal receptacle of *Haplotropis brunneriana* (Orthoptera)^83^. Visualization and segmentation analyses of FIB-SEM data showed EVs on the apical surface in virgin and mated distal seminal receptacles. EV spatial distribution and heterogeneity changed post-mating. EVs were also detected in the seminal receptacle lumen, suggesting interaction with sperm to maintain their viability in storage.

Future studies will decipher whether the distal seminal receptacle epithelium secretes EVs to the lumen, which are taken up by sperm, and if the epithelium takes up EVs released by sperm in storage. Additional studies will uncover the role that these EVs play in maintaining sperm in storage. We also need to understand the mechanism by which the corrugated intima and the peristaltic contractions of the seminal receptacle muscular layer mediate flow and pressure inside the lumen.

Finally, our results point to the importance of studying organ morphology at high spatial resolution using various microscopy modalities, applying a correlative approach and image analysis. This combination of data gave us a more profound understanding of the anatomy of the lower reproductive tract and distal seminal receptacle than could be obtained by any of the microscopy modalities alone. Furthermore, such multimodal combinations reflected on the complexities of communication networks and possible mechanisms enabling sperm to survive for extended periods within female storage organs.

## Methods

### Flies

Wild-type Canton-S flies were used for all microscopy analyses. Flies were held in a 12 h light/dark cycle at 23 ± 2 °C. Upon eclosion, females and males were collected on ice and held separately until 3 days of age. To visualize sperm in the seminal receptacle, females were mated with *don juan (dj)*-GFP^84^ or *protamine (pro)*-GFP males (provided by the E. Arama lab).

### Developing a staining method to generate enhanced contrast image visualization by microCT

To enhance contrast in the lower reproductive tract, we used Iodine crystal (I2) (#376558, Merck), Phosphotungstic acid (#12501-23-4, AnalaR), Osmium tetroxide (#19152, EMS), and a combination of I2, Tannic acid (#1401-55-4, Merck) and Ruthenium red (#00541, Sigma-Aldrich), which are regularly used for other soft tissues and/or for other microscopy applications (for additional details see Supplementary Table 1).

### Sample preparation

For all assays, unless described otherwise, virgin 3-day-old females were placed singly in vails with a 3-day-old virgin male and the pairs were observed until mating was initiated. At the end of mating, females were aspirated into fresh vials and held for 3–5 h before being placed on ice for dissection.

#### Confocal microscopy

Female reproductive tracts were dissected in PBS (0.85% NaCl, 1.4 mM KH2 PO4, 8 mM Na2 HPO4, pH 7.4) on ice and immediately fixed in: 1) 4% formaldehyde (FA) (#157-4, EMS) in PBS for 1 h at room temperature, for non-embedded tissue, or 2) 2% FA and 3% glutaraldehyde (#16020, EMS) in 0.1 M Cacodylate buffer and 5 mM CaCl2 (pH = 7.4) for 1 h and then overnight at 4 °C, for embedded tissue. Following washing in PBS or 0.1 M Cacodylate buffer (non-embedded/embedded respectively) for 3 × 5 min, each sample was arranged on a small triangular piece of either plastic sheeting (P.P.C film) for the non-embedded correlation, or on a similar shaped piece of Aclar film (#01858-CF, SPI) for the embedded correlation. Both sheet types were coated with 0.01% polylysine (#4707, Sigma-Aldrich). The female reproductive tract was positioned on the sheet and allowed to adhere for 10 s with a minimal amount of buffer surrounding the sample. The sample was then re-immersed in fresh PBS buffer and transferred to the confocal microscope for imaging.

#### Correlative confocal and micro computed tomography (microCT)

##### Non-embedded tissue

After confocal scanning, the plastic sheets containing the samples were transferred to a 96-well plate for dehydration in a graded series of ethanol (30, 50, 70, 90, 95, 3 × 100%) washes, 10 min each, at room temperature. Samples were then stained with a mixed contrasting dye [1% I2 and 1% Tannic acid in 100% ETOH] for 48 h at 4 °C. Before imaging, the samples were washed in fresh 100% ETOH 2 × 10 min.

##### Embedded tissue

Following confocal scanning, each sample on the Aclar film was post-fixed with OsO4 solution (1% OsO4, 0.5% Potassium dichromate, 0.5% Potassium hexacynoferrate, in 0.1 M Cacodylate buffer) for 1 h at 25 °C. Samples were then washed 2 × 5 min in 0.1 M Cacodylate buffer followed by 4 × 3 min washes in DDW, stained with 2% Uranyl acetate in DDW, and covered with aluminum foil for 1 h. Thereafter, the samples were washed 4 × 5 min with DDW and dehydrated in a graded series of ethanol (30, 50, 70, 90, 95, 3 × 100%) 10 min each on ice. Samples were then incubated with propylene oxide (#00236, Polysciences Inc.) 3 × 5 min at room temperature, and transferred to 50% followed by 75% Epon (#08791-500, Polyscience Inc.) in propylene oxide, each overnight. Samples were then left in a hood for 4 h to allow the propylene oxide to evaporate, and then infiltrated with 100% Epon overnight and fresh Epon 3X2h. All the above steps were done without agitation. Finally, samples were embedded in a flat mold and polymerized at 60 °C for 48 h.

#### MicroCT

Non-embedded samples, wet, soft tissue on a plastic sheet, were securely placed in a plastic pipette tip. The tip was heat-sealed, filled with 100% ETOH, the triangular plastic sheet was secured in the pipette tip, which was then sealed with Parafilm^85,86^. The embedded sample block was attached to a standard holder.

#### Correlative microCT and focused ion-beam scanning electron microscopy (FIB-SEM)

Low-magnification microCT tomography (4×) of the epon-embedded samples guided the FIB-SEM milling (see Fig. 3). Based on the microCT measurements, the blocks were trimmed from the top to the desired area using an ultramicrotome (Leica UC7). A protective platinum patch (5–7 nm; Q150T ES Quorum) was deposited onto the exposed block surface above the ROI using ion beam- induced CVD deposition at 30 keV, 0.46 Na, and the sample was viewed under SEM (Helios 600 Dual Beam, FEI). This patch served to increase sample conductivity and to reduce charging effects during FIB milling. A 25 ×35 μm trench was milled just in front of the ROI at 20 nA. Following SEM imaging, a second microCT scan was performed at a higher magnification and resolution (20×), and the location of the SEM block surface image plane was located in the 3D tomogram. This location was used as a vector for the precise measurement of VOI for the FIB-SEM milling starting point. Polishing of the exposed surface and serial milling was performed using a lower ion beam current of 0.7 nA.

#### Resin section for scanning transmitted electron microscopy (STEM)

Samples were embedded as described above for FIB section embedding, with the additional of first flat embedding the female reproductive tract between two Aclar films. Following curing and excision of the embedded tract, samples were re-embedded in a flat mold. Embedded samples were sectioned using an ultramicrotome (RMC, PowerTome PT-XL, Boeckeler, USA).

#### Cryostat sections for SEM

Unmated and mated females were fixed for 1 h in 4% FA after the removal of legs and wings, washed 3 × 5 min in PBS (0.85% NaCl, 1.4 mM KH2 PO4, 8 mM Na2 HPO4, pH 7.4) and incubated in 25% sucrose overnight at 4 °C. Flies were briefly washed in PBS before embedding in O.C.T (#4583, Tissue-Tek) and freezing on a LN2 cooled metal block. Using a cryostat (Leica CM1950), 20 µm sections were collected on super plus glass slides (Thermo Scientific) and coated with 1 nm AuPd (Quorum Q 150 T ES, UK).

### Microscopy

#### Confocal microscopy

For imaging, the plastic sheet with the reproductive tract was placed on a Glass Bottom Dish (#200350, SPL Life Sciences). Images were taken either with the sample facing the glass, or through the plastic sheet. Reproductive tracts were viewed with a Leica SP8 laser scanning confocal microscope using Leica HC PL APO CS 20 × 0.7 N.A and HC PL APO CS2 63 × 1.2 N.A water objectives. The laser used was EX 488 EM 520-540 nm for GFP. Optical z-sections from various focal planes of 1 µm sections at 20× and 0.3 µm sections at 63× of each reproductive tract region, were collected and projected as a reconstructed 3D image using LAS AF Leica software.

Protamine-GFP sperm visualization in the distal seminal receptacle was carried out using a 20× objective in an inverted Zeiss 510 confocal microscope with 488 nm excitation and 520–540 nm emission.

#### MicroCT

Scans were acquired using a microXCT-400 (Zeiss Xradia, California, USA). For non-embedded samples, tomograms were acquired at 20x magnification, obtained from 1000 projections, with exposure time of 7 s (acquired over 180°) at 40 kV and 200 µA. The final voxel size was 0.66 µm. Embedded samples were scanned at 4× or 20× magnification, depending on the purpose of scan. For the low magnification (4×) 500 projections, 4 s exposures were acquired over 180°, at 40 kV and 200 µA. The final voxel size was 5.5 µm. After sectioning the block, a high-resolution microCT scan was run at 20× magnification. Tomographic images were obtained from 1000 projections, with an exposure time of 10 s (acquired over180°) at 40 kV and 200 µA. The final voxel size was 0.75 µm.

#### FIB-SEM

For volumetric visualization of the distal seminal receptacle internal structure, we used the serial surface view (SSV) method^87^ performed on the Helios-600 Dual Beam instrument (Thermo Fisher Scientific, Oregon, USA). SEM images were sequentially collected at 2.5 kV, 340 pA, at “immersion lens” mode with TLD detector, spaced 25 nm apart. The process took 3 days. The dimensions of the acquired datasets varied, as specified below in Table 1.

**Table 1:** The dimensions of the SEM acquired datasets in virgin and mated samples.

Sample	Virgin	Mated
SEM image pixel size, nm	14 × 14	13 × 13
Number of slices	560	640
Spacing distance, nm	25	25
Presented box dimensions, µm	14 × 14 × 14	23 × 22 × 16

#### STEM

Resin sections of the seminal receptacle were viewed with a Jeol JSM 7800F with a Deben Gen5 bright and dark field retractable STEM detector (Deben, UK) at 30 KV.

#### SEM

Cryostat sections were viewed with a Jeol JSM 7800F with a LED detector (ET SE detector) at 2 KV.

### Image analysis

#### FIB

The acquired FIB-SEM datasets were pre-processed prior to performing the segmentation and correlation steps with Fiji/Image J (NIH, USA) software. First, the image stacks were aligned using the “StackReg” plugin. The images were then digitally filtered to improve visualization of structural details. The raw images were initially filtered using the FFT bandpass filter tool in order to remove artifacts produced during the milling process (curtaining effect). Contrast enhancement was obtained using the (CLAHE) process. The images’ gray levels through the stack were equalized with the bleach correction process using the histogram matching correction method.

3D visualization for the FM-microCT-FIB correlative models was performed with Amira software (Thermo Fisher Scientific, USA). A VolRen module was used for the FM and microCT data, and three Ortho-Slice projections presented FIB-SEM datasets. Correlation was performed manually, in accordance with the morphologic landmarks, using Transform Editor of Amira SW.

3D structural details of the localized segments of the seminal receptacle were visualized using Dragonfly software, developed by Object Research Systems (ORS), (Montreal, QC, Canada). FIB-SEM data was used to perform segmentation of the lower reproductive tract, including extracellular vesicles. Segmentation was carried out manually with the adaptive 3D brush feature. Video files were composed and registered with the Video Maker tool. Vesicles size analyses were conducted using the Data Statistical Property feature.

#### X-Ray image acquisition and analysis

Raw images were uploaded to Avizo 9.2.0 (VSG, USA) for 3D model reconstruction. Since microCT images were used as reference for positioning the FIB-SEM beam, we kept the image processing to a minimum. No digital filters or any other image processing operations were applied. The Volume Rendering function of Amira software (2019.3) was used to display 3D microCT images. The slice function of Amira was used to represent the inner sections through the samples. Volume Rendering was also performed using CTVox software (SkyScan, Bruker). Slicing was spatially oriented in order to optimally reveal the inner structure of the samples.

A ‘.tiff’ sequence of a mated female was exported from Avizo for further analysis with ilastik’s auto-context workflow (version 1.3.3post3)^70^. To achieve higher hardware usage efficiency, the ‘.tiff’ sequence was converted to the ‘.h5’ file format, which runs faster in ilastik. With the autocontext workflow, a ground-truth annotation and training were made in two steps for the background (BG), outer lamella (OL), and inner lamella (IL). In the first training step, the image data were labeled into three classes, as described above. In the second training round, the data were labeled BG and OL (ilastik project, ‘.ilp’ copy1), and later BG and IL (copy 2). Two copies of the ilastik model were made to avoid conflicts in pixel prediction; in that way, only the first training annotations are saved in the ‘.ilp’ file, and later the user can make multiple predictions for the same data with greater accuracy. The results of both predictions, copy 1 and 2, were exported as the ‘stage 2 probabilities’ 16-bit ‘.tiff’ sequence for further merging, and 3D viewing of the OL and IL datasets, in Icy (version 2.3.0.0)^88^. The last step included Imaris’s surface, spots, and animation modules for video creation (version 9.0.2) (Oxford Instruments, England).

### Statistics and reproducibility

To assess differences in vesicle diameter, we divided all vesicles into ten size categories and compared the mated versus virgin outcome frequencies. Pearson’s Chi-Square test of row-column dependency was used for analysis. Pearson’s χ2 test: χ2 = 273.30, *p* < 0.0001 indicated independence between mated and virgin samples. Kendall’s τb and τc supported this test by showing ranking differences between mated and virgin vesicle diameters (τ = −15.15, *p* < 0.0001). The analyses were performed using SPSS V26.0.

### Reporting summary

Further information on research design is available in the Nature Portfolio Reporting Summary linked to this article.

### Supplementary information

SUPPLEMENTAL MATERIAL

Description of Additional Supplementary Files

Supplementary Data 1

Supplementary Data 2

Supplementary Video 1

Supplementary Video 2

Supplementary Video 3

Supplementary Video 4

Supplementary Video 5

Supplementary Video 6

Reporting Summary

## Data Availability

All other data are available from the corresponding author upon request. The raw data for Fig. 5e and Supplementary Figure 2 can be found in Supplementary Data 1 and 2 respectively.
