# Supplementary material for: Three-dimensional correlative microscopy of the Drosophila female reproductive tract reveals modes of communication in seminal receptacle sperm storage
Source: Commun Biol. 2024 Feb 6;7:155. doi: 10.1038/s42003-024-05829-y (PMC10847118; doi:10.1038/s42003-024-05829-y)
Supplement: Supplementary file 1 — SUPPLEMENTAL MATERIAL [file 42003_2024_5829_MOESM1_ESM.pdf]

Three-dimensional correlative microscopy of the Drosophila female reproductive tract reveals modes of communication in seminal receptacle sperm storage

Einat Zelinger [1,2,#], Vlad Brumfeld [4,#]. Katya Rechav [4,#], Daniel Waiger [2], Tally Kossovsky[2], Yael Heifetz [1*]

[1]Department of Entomology, The Hebrew University, Rehovot, Israel, [2]Center for Scientific Imaging, The Hebrew University, Rehovot, Israel, [3]Chemical Research Support Department, Weizmann Institute, Rehovot, Israel; [#]equal contribution,[*]corresponding author

This PDF file includes:

Supplementary Note 1
Supplementary Tables 1 and 2
Supplementary Figures 1 and 2
Supplementary Legends for Videos and Supplementary Data

Other supplementary materials for this manuscript include:

Videos 1 to 6
Supplementary Data 1
Supplementary Data 2

SUPPLEMENTARY NOTE 1

Combination of contrasting stains, iodine, and tannic acid enhanced visualization using microCT

The success of microCT scanning is heavily dependent on preparation and tissue contrast during visualization with the x-ray beam. Since high resolution spatial imaging using microCT requires enhanced contrast of the soft tissues examined, we tested different contrasting stains and their combinations (**Supplementary Figure 1, Supplementary Table 1**). Inorganic iodine (I2) and phosphotungstic acid (PTA) are the most common stains used for soft, low electron dense tissues; their high atomic number and ability to absorb x-rays improve contrast of a given specimen[1-3]. Staining the Drosophila reproductive tract with each of the contrasting stains gave good external contrast, but resolution of the internal tissues was poor. For example, iodine or phosphotungstic acid stained the internal tissues of the tract, but contrast was too low to distinguish among different anatomical features of the lower reproductive tract (**Supplementary Figure 1a, a', b, b', respectively**). Combining tannic acid and iodine enhanced the contrast and resolution of the external and internal tissues, but we could not detect the sperm inside the seminal receptacle (**Figure 1d, Figure 2**). To further increase resolution, we added ruthenium red to the iodine and tannic acid; this dye is used in plants or for cartilage matrix visualization via x-ray[4,5]. This combination did not, however, improve contrast of the internal structures (**Supplementary Figure 1c, c'**). The use of osmium tetroxide, which is part of the embedded tissue preparation[1,6], alone, or in combination with iodine and tannic acid, increased the internal contrast but this improvement was insufficient for detailed segmentation (**Supplementary Figure 1d, d', e, e', respectively**). Although we were unable to image sperm in storage, we found the combination of iodine and tannic acid to give the best contrast. Going forward, this combination was thus used as a contrasting stain for visualization with microCT. In addition to applying the combination of stains, each sample was arranged on a small triangular piece of plastic sheeting which stabilized the orientation of the sperm storage organs and enhanced the clarity of the imaging.

SUPPLEMENTARY TABLES

Supplementary Table 1. Different stains used for visualizing the female lower reproductive tract with microCT

Type of Stain	Concentration used	Protocol
Iodine crystal (I2)	2% I2 in 100% ETOH	Incubate with stain for 48h at 4^0C. Wash for 10min in ETOH. Transfer to fresh ETOH until scanning
Phosphotungstic acid (PTA)	1% PTA in 100% ETOH	Incubate for 48h at 4^0C. Wash for 10min and transfer to fresh ETOH until scanning
I2, tannic acid (Ta) and Ruthenium red (RR)	1% I2, 1% Ta and 1% RR in 100% ETOH	Add 1% RR to the fixation mix as in microCT for non-embedded tissue. After dehydration, incubate with I2 and Ta for 48h at 4^0C. Wash for 10min in ETOH. Transfer to fresh ETOH until scanning
Osmium tetroxide (OsO4)	1% OsO4, 0.5% Potassium dichromate, 0.5% Potassium hexacynoferrate, in 0.1 M Cacodylate buffer and 2% Uranyl acetate.	Sample preparation as in microCT for embedded tissue (see material and methods) until reaching the 100% ETOH step. Keep in ETOH until scanning
I2, Ta and OsO4	1% OsO4 (as above), 1% I2 and 1% Ta in 100% ETOH	Sample preparation as in microCT for embedded tissue including OsO4 until the 100% ETOH step. Incubate with I2 and Ta mix for 48h or longer at 4^0C. Wash for 10min in ETOH. Transfer to fresh ETOH until scanning
OsO4	1% OsO4 (as above)	Sample preparation for embedded tissue (see material and methods). Samples were scanned through the block.

All samples were prepared as described in the material and method (section microCT samples preparation for non-embedded) or as stated otherwise. All tubes were covered with foil during staining.

Supplementary Table 2. Advantages and disadvantages of using different combination of correlative Light microscopy, Microcomputed tomography and Focused Ion Beam-Scanning Electron microscopy.

**Multi modal volume Combination**	**Advantage**	**Disadvantage**
LM<br>microCT	High resolution of internal *in-situ* localization within the whole organ at the tissue level<br><br>Non-destructive<br><br>The sample remains fully hydrated (depends on the contrast stain) and requires only a slight processing<br><br>Light microscopy preparation can be followed by embedding<br><br>Can serve as a good screening tool prior to embedding the sample	Less suitable for *in-situ* high resolution at the cellular level
microCT<br>FIB-SEM	microCT is used as a ground map to guide the acquisition volume by the FIB milling<br><br>Combination of high repeatability and precision with high cellular resolution<br><br>Suitable for external features or highly distinctive internal localizations	Translucent features will not be detected without staining
LM<br>FIB-SEM	Suitable for samples in which the region of interest is highly distinct or big enough to be detected for FIB milling	Localization specificity is limited, small scale region of interest, or subcellular structures cannot be detected
LM<br>microCT<br>FIB-SEM	Highly specific internal in-situ localization within the whole organ<br><br>LM- microCT is used as a ground map for the FIB milling<br><br>High repeatability and precision with high cellular resolution	

LM, Light Microscopy (Confocal).
microCT, Microcomputed tomography
FIB-SEM, Focused Ion Beam-Scanning Electron Microscopy

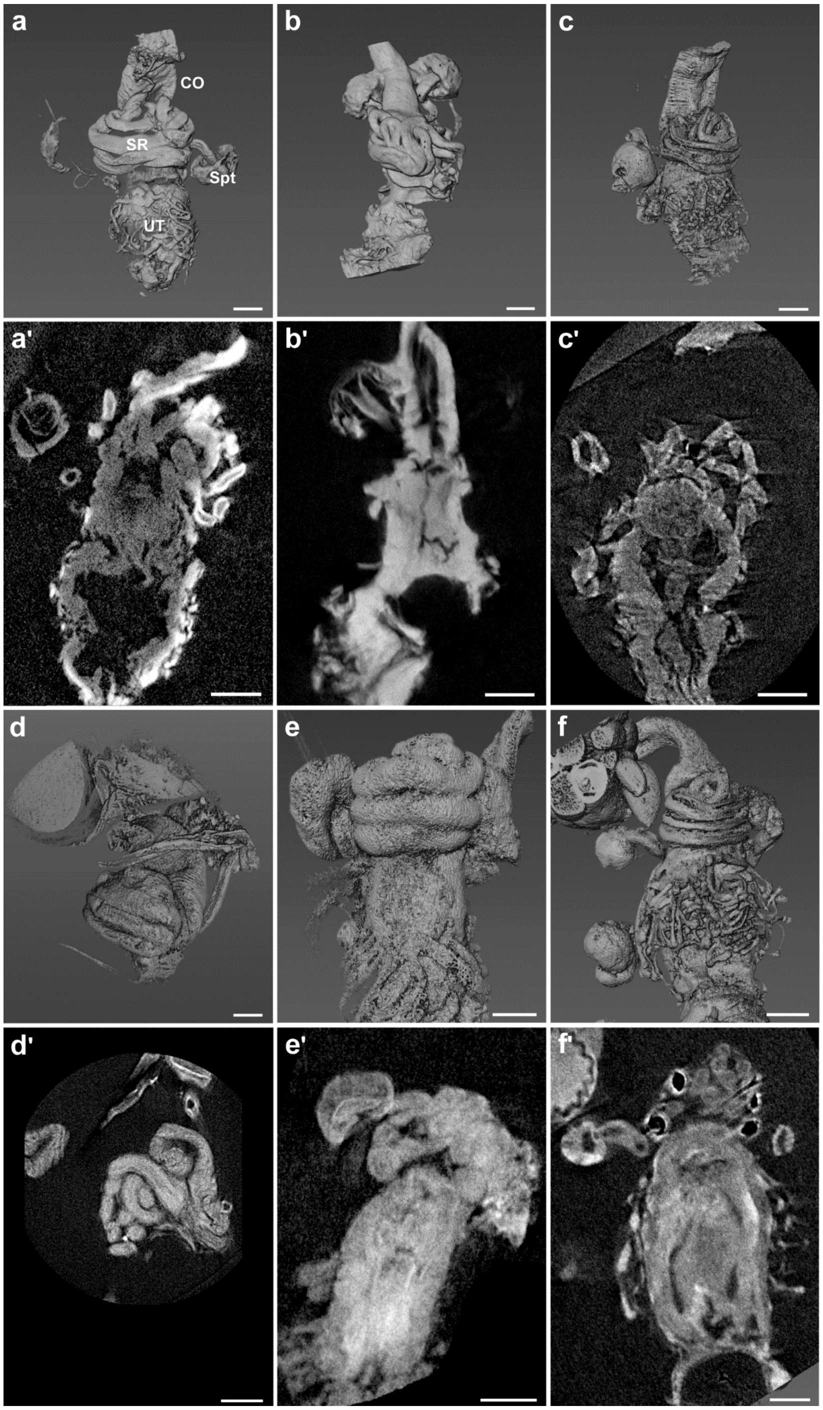

Supplementary Figure 1. **Examination of different contrast staining to enhance microCT visualization.** microCT 3D images **(capital letter)** and its counterpart 2D section **(capital letter ')**. **(a, a')** Iodine and PTA staining (1% I2 in 100% ETOH) enabled a good external volume view relative to the other stains used. However, the internal structures were poorly preserved. SR, seminal receptacle; CO, common oviduct; Spt, spermatheca; UT, uterus. **(b, b')** 1% PTA in 100% ETOH. **(c, c')** Ruthenium red 1% in 100% ETOH. **(d, d')** Osmium 1% in cacodylate buffer and I2 1% in ETOH. **(e, e')** Tannic acid 1% in ETOH. Scale bar=50 µm. **(f, f')** 1% Osmium in resin block; scale bar=70 µm.

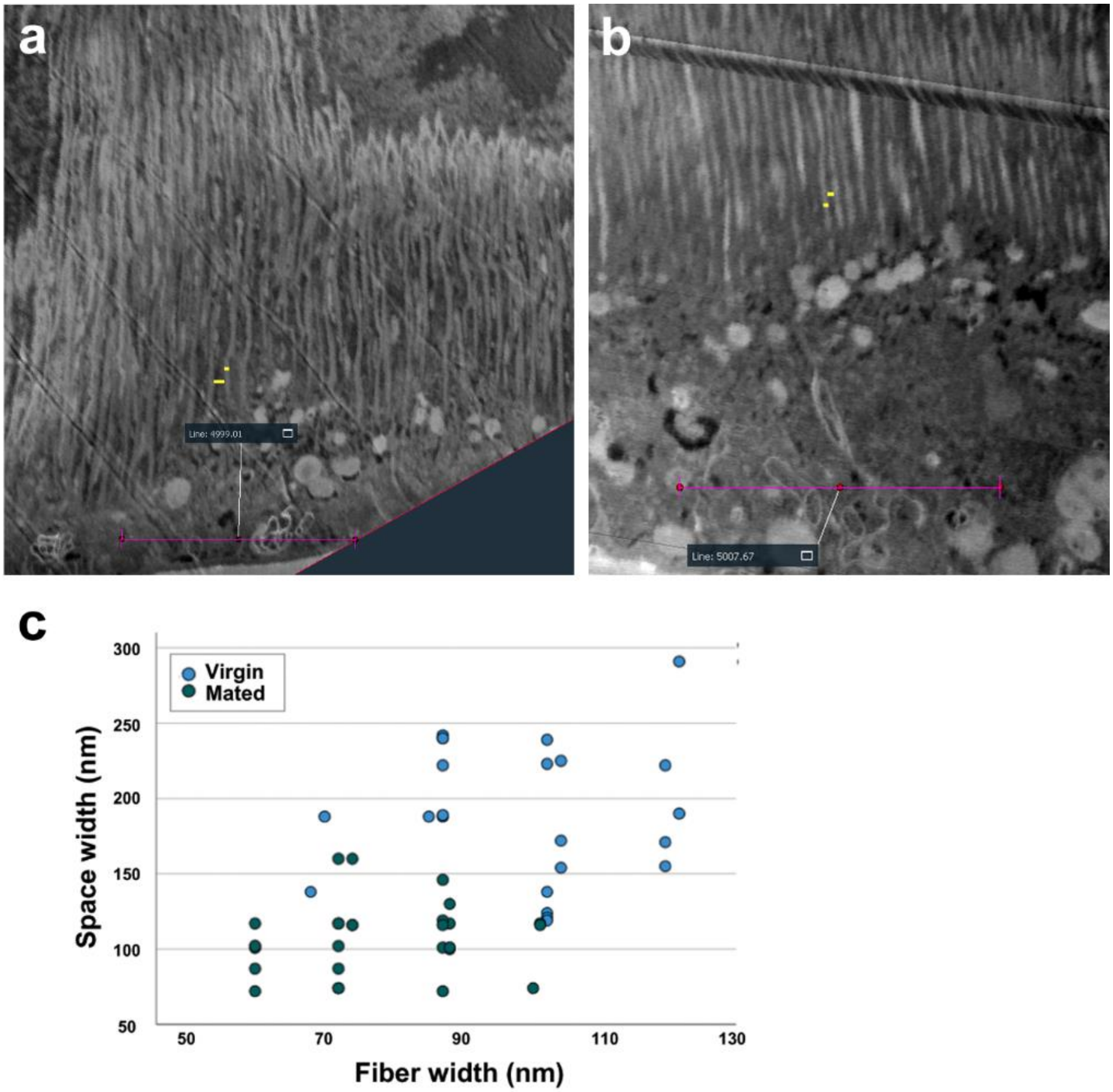

Supplementary Figure 2. **Organization of virgin and mated intima fiber layer of the distal seminal receptacle.** Measurements of fiber width and the space between fibers were done using images of virgin **(a)** and mated **(b)** aligned to their physical axis; scale bar=5μm. **(c)** Graph showing the distribution of fiber width and the width of the space between fibers of virgin and mated intima. Blue circles represent virgin; green circles represent mated.

SUPPLEMENTARY VIDEOS

Supplementary Video 1. A microCT movie showing the morphology of mated internal seminal receptacle and uterus.

Supplementary Video 2. Segmentation of the lower reproductive tract shows thick multilayer uterine circular muscle fibers (purple) and infoldings of the uterine epithelial cells (cyan) (see also Figure 2).

Supplementary Video 3. A video showing the correlative work flow and integration for milling the precise region of interest using FIB-SEM (see also Figure 3).

Supplementary Video 4. Three-dimensional FIB-SEM projection and segmentation movie showing virgin distal seminal receptacle. Corrugated intima facing the lumen (blue) and a fiber layer (green) facing the microvilli, vesicles (pink), septate junction (pale pink), muscle (brownish) (see also Figure 4).

Supplementary Video 5. Three-dimensional FIB-SEM projection and segmentation movie showing mated distal seminal receptacle. Sperm in the lumen (dark yellow), corrugated intima facing the lumen (blue) and a fiber layer (green) facing the microvilli, vesicles (purple, pink), septate junction (pale pink), muscle (yellow) (see also Figure 4).

Supplementary Video 6. A video showing segmentation of the intima fiber layer highlighting the heterogeneous population of EVs on the apical region of the distal seminal receptacle (See also Figure 5).

SUPPLEMENTARY DATA

Supplementary Data 1. Raw data of Figure 5e.

Supplementary Data 2. Raw data of Supplementary Figure 2.

REFERENCES

1. Metscher, B. D. MicroCT for Comparative Morphology: Simple Staining Methods Allow High-Contrast 3D Imaging of Diverse Non-Mineralized Animal Tissues. *BMC Physiol*. **9**, 11. (2009).

2. Heimel, P. *et al.* Iodine-Enhanced Micro-CT Imaging of Soft Tissue on the Example of Peripheral Nerve Regeneration. *CMMI*. **2019**, 7483745 (2019).

3. Lesciotto, K. M. *et al.* Phosphotungstic Acid-Enhanced microCT: Optimized Protocols for Embryonic and Early Postnatal Mice. *Dev. Dyn.* **249**, 573-585 (2020).

4. Retamales, H. A. & Scharaschkin, T. A. Staining Protocol for Identifying Secondary Compounds in Myrtaceae. *Appl. Plant Sci.* **2**, 1400063 (2014).

5. Gabner, S. *et al.*. The Visible Skeleton 2.0: Phenotyping of Cartilage and Bone in Fixed Vertebrate Embryos and Foetuses Based on X-ray microCT. *Dev*. **147**, dev187633 (2020).

6. Van den Boogert, T. *et al.* Optimization of 3D-Visualization of Micro-Anatomical Structures of the Human Inner Ear in Osmium Tetroxide Contrast Enhanced Micro-CT Scans. *Front. Neuroanat.* **12**, 41 (2018).
