# Supplementary material for: Three-dimensional correlative microscopy of the Drosophila female reproductive tract reveals modes of communication in seminal receptacle sperm storage
Source: Commun Biol. 2024 Feb 6;7:155. doi: 10.1038/s42003-024-05829-y (PMC10847118; doi:10.1038/s42003-024-05829-y)
Supplement: Supplementary file 2 — Description of Additional Supplementary Files [file 42003_2024_5829_MOESM2_ESM.pdf]

Description of Additional Supplementary Files

File name: Supplementary Data 1
Description: Raw data of Figure 5e.

File name: Supplementary Data 2
Description: Raw data of Supplementary Figure 2.

File name: Supplementary Video 1
Description: A microCT movie showing the morphology of mated internal seminal receptacle and uterus.

File name: Supplementary Video 2
Description: Segmentation of the lower reproductive tract shows thick multilayer uterine circular muscle fibers (purple) and infoldings of the uterine epithelial cells (cyan) (see also Figure 2).

File name: Supplementary Video 3
Description: A video showing the correlative workflow and integration for milling the precise region of interest using FIB-SEM (see also Figure 3).

File name: Supplementary Video 4
Description: Three-dimensional FIB-SEM projection and segmentation movie showing virgin distal seminal receptacle. Corrugated intima facing the lumen (blue) and a fiber layer (green) facing the microvilli, vesicles (pink), septate junction (pale pink), muscle (brownish) (see also Figure 4).

File name: Supplementary Video 5
Description: Three-dimensional FIB-SEM projection and segmentation movie showing mated distal seminal receptacle. Sperm in the lumen (dark yellow), corrugated intima facing the lumen (blue) and a fiber layer (green) facing the microvilli, vesicles (purple, pink), septate junction (pale pink), muscle (yellow) (see also Figure 4).

File name: Supplementary Video 6
Description: A video showing segmentation of the intima fiber layer highlighting the heterogeneous population of EVs on the apical region of the distal seminal receptacle (See also Figure 5).
