# Supplementary material for: Three-dimensional correlative microscopy of the Drosophila female reproductive tract reveals modes of communication in seminal receptacle sperm storage
Source: Commun Biol. 2024 Feb 6;7:155. doi: 10.1038/s42003-024-05829-y (PMC10847118; doi:10.1038/s42003-024-05829-y)
Supplement: Supplementary file 11 — Reporting Summary [file 42003_2024_5829_MOESM11_ESM.pdf]

nature research

Corresponding author(s): Yael Heifetz

Last updated by author(s): Feb 16, 2023

Reporting Summary

Nature Research wishes to improve the reproducibility of the work that we publish. This form provides structure for consistency and transparency in reporting. For further information on Nature Research policies, see our Editorial Policies and the Editorial Policy Checklist.

Statistics

For all statistical analyses, confirm that the following items are present in the figure legend, table legend, main text, or Methods section.

n/a	Confirmed	
☐	☒	The exact sample size (*n*) for each experimental group/condition, given as a discrete number and unit of measurement
☒	☐	A statement on whether measurements were taken from distinct samples or whether the same sample was measured repeatedly
☐	☒	The statistical test(s) used AND whether they are one- or two-sided<br>*Only common tests should be described solely by name; describe more complex techniques in the Methods section.*
☒	☐	A description of all covariates tested
☒	☐	A description of any assumptions or corrections, such as tests of normality and adjustment for multiple comparisons
☐	☒	A full description of the statistical parameters including central tendency (e.g. means) or other basic estimates (e.g. regression coefficient) AND variation (e.g. standard deviation) or associated estimates of uncertainty (e.g. confidence intervals)
☐	☒	For null hypothesis testing, the test statistic (e.g. F, t, r) with confidence intervals, effect sizes, degrees of freedom and P value noted<br>*Give P values as exact values whenever suitable.*
☒	☐	For Bayesian analysis, information on the choice of priors and Markov chain Monte Carlo settings
☒	☐	For hierarchical and complex designs, identification of the appropriate level for tests and full reporting of outcomes
☒	☐	Estimates of effect sizes (e.g. Cohen's d, Pearson's r), indicating how they were calculated

Our web collection on statistics for biologists contains articles on many of the points above.

Software and code

Policy information about availability of computer code

Data collection	*Provide a description of all commercial, open source and custom code used to collect the data in this study, specifying the version used OR state that no software was used.*
Data analysis	*Provide a description of all commercial, open source and custom code used to analyse the data in this study, specifying the version used OR state that no software was used.*

For manuscripts utilizing custom algorithms or software that are central to the research but not yet described in published literature, software must be made available to editors and reviewers. We strongly encourage code deposition in a community repository (e.g. GitHub). See the Nature Research guidelines for submitting code & software for further information.

Data

Policy information about availability of data

All manuscripts must include a data availability statement. This statement should provide the following information, where applicable:

- Accession codes, unique identifiers, or web links for publicly available datasets
- A list of figures that have associated raw data
- A description of any restrictions on data availability

The raw data of the images is available upon request

Field-specific reporting

Please select the one below that is the best fit for your research. If you are not sure, read the appropriate sections before making your selection.

☒ Life sciences ☐ Behavioural & social sciences ☐ Ecological, evolutionary & environmental sciences

For a reference copy of the document with all sections, see nature.com/documents/nr-reporting-summary-flat.pdf

Life sciences study design

All studies must disclose on these points even when the disclosure is negative.

Sample size	In our experimental design, we included 2 independent factors: time (24 hours post-mating) and mating status (virgin, mated). For the microCT experiments we used 14 flies; FIB-SEM we used 3 flies; TEM we used 6 flies, and for Confocal we used at least 10 flies. For each imaging experiment flies were taken randomly from each time and mating status.
Data exclusions	No Data were excluded
Replication	Replication was performed by analysing different WT flies and flies from different crosses. The variability observed was the result of this, and in spite of this variability the effects were consistent.
Randomization	The tissue samples were imaged and analysed randomly but under the same parameters.
Blinding	Blinding was not possible as the experimental groups required to have a controlled time post-mating in order to set the parameters of imaging equal for all the experimental groups.

Reporting for specific materials, systems and methods

We require information from authors about some types of materials, experimental systems and methods used in many studies. Here, indicate whether each material, system or method listed is relevant to your study. If you are not sure if a list item applies to your research, read the appropriate section before selecting a response.

Materials & experimental systems

n/a	Involved in the study	
☐	☒	Antibodies
☒	☐	Eukaryotic cell lines
☒	☐	Palaeontology and archaeology
☐	☒	Animals and other organisms
☒	☐	Human research participants
☒	☐	Clinical data
☒	☐	Dual use research of concern

Methods

n/a	Involved in the study	
☒	☐	ChIP-seq
☒	☐	Flow cytometry
☒	☐	MRI-based neuroimaging

Antibodies

Antibodies used	We used the following stains: Calcofluor White Stain (Sigma-Aldrich, Cat. No. 18909); Iodine crystal (#376558, Merck); Phosphotungstic acid (#12501-23-4, AnalaR); Osmium tetroxide (#19152, EMS); Tannic acid (#1401-55-4, Merck); Ruthenium red (#00541, Sigma-Aldrich)
Validation	Fluorescent or other imaging criteria cut-offs were set relative to the control.

Animals and other organisms

Policy information about studies involving animals; ARRIVE guidelines recommended for reporting animal research

Laboratory animals	Drosophila melanogaster. Both male and female flies were 3 days old. The lines employed and husbandry are detailed in the methods.
Wild animals	*Provide details on animals observed in or captured in the field; report species, sex and age where possible. Describe how animals were caught and transported and what happened to captive animals after the study (if killed, explain why and describe method; if released, say where and when) OR state that the study did not involve wild animals.*
Field-collected samples	*For laboratory work with field-collected samples, describe all relevant parameters such as housing, maintenance, temperature, photoperiod and end-of-experiment protocol OR state that the study did not involve samples collected from the field.*

Ethics oversight | Flies do not require the approval from a ethics committee.

Note that full information on the approval of the study protocol must also be provided in the manuscript.
